# TraumaLink: A Community-Based First-Responder System for Traffic Injury Victims in Bangladesh

**DOI:** 10.9745/GHSP-D-21-00537

**Published:** 2022-08-30

**Authors:** Jon Moussally, Arup Chandra Saha, Susan Madden

**Affiliations:** a Harvard T.H. Chan School of Public Health, Department of Global Health and Population, Boston, MA, USA.; b TraumaLink, Dhaka, Bangladesh.

## Abstract

**Introduction:**

Road traffic injuries are a rapidly growing epidemic in low- and middle-income countries (LMICs). However, many countries lack formal prehospital emergency medical services, often leaving victims without access to first aid when it can be most effective in preventing death or disability.

**Methods:**

To address the lack of a dedicated prehospital emergency medical system in Bangladesh, we developed TraumaLink, a community-based network of volunteer first responders for traffic injury victims. The service uses an emergency hotline number and 24-hour call center with local first responders who are trained in basic trauma first aid, given essential medical supplies, and dispatched to crash scenes through mobile phone text message notifications. We designed the training curriculum to teach simple lifesaving skills that people with any level of education and no prior medical background could learn and perform. We retrospectively analyzed data originally collected for quality monitoring and evaluation to provide a descriptive analysis of the program’s impact.

**Results:**

During the first 6 years, operations were expanded from a 14-km section of 1 highway to 135 km on 3 national highways, and free care was provided to 3,119 patients involved in 1,544 crashes. All calls to the service received a response, and in 88% of cases, first responders were at the scene in 5 minutes or less. Most patients were young adult men, and 76% of victims transported to the hospital arrived there within 30 minutes of the crash. Assessments of injury severity at the accident scene aligned closely with patient dispositions, reflecting the accuracy of these triage decisions.

**Conclusion:**

The strong community support and rapid, reliable volunteer responses suggest that this flexible and scalable model could be expanded throughout Bangladesh and adapted for other LMICs that face similar challenges with traffic injury victims.

## Abstract (precis)

A community-based network of trained volunteer layperson first responders in Bangladesh provided rapid and reliable on-scene trauma care to traffic injury victims, free of charge.

## Teaser

**Key Findings:**

- TraumaLink established a community-based emergency response model to address barriers in rapidly responding to road traffic accidents, accurately triaging patients, providing first aid, and transporting victims to hospitals in a timely manner to reduce crash-related deaths and disability.
- Communities showed a high level of acceptance and dedication to the program, evidenced by high volunteer recruitment and retention rates, and a 100% incident response rate.
- The program has expanded from a 14-km section of 1 highway to 135 km on 3 national highways.

**Key Implications:**

- Policy makers and stakeholders in Bangladesh may consider expanding a volunteer-based, emergency response system on a larger scale and broadly integrating it with police and fire services to help manage traffic accidents.
- Other low- and middle-income countries could adapt this service model to help manage similar challenges with traffic injury victims.

## INTRODUCTION

Road traffic injuries (RTIs) are a rapidly growing epidemic in low- and middle-income countries (LMICs) but often get less recognition and attention than other health care challenges.^1^^–^^8^ In 2016, there were an estimated 1.35 million fatalities from RTIs worldwide, with the burden falling most heavily on LMICs. Although these countries have about 60% of the world’s vehicles, LMICs account for 93% of all traffic fatalities worldwide,^9^ with a lack of rapid access to prehospital medical care and transportation commonly playing an important role in this excess mortality.^10^^–^^12^ The association between these deaths and the lack of timely response is to be expected given that traumatic injuries are exquisitely time-sensitive, reinforcing the concept of the “golden hour”—which holds that patient outcomes are improved if they receive definitive care within 1 hour of sustaining their injuries. The urgency of this public health crisis was well evidenced in the United Nations Sustainable Development Goal 3.6, adopted with the intent of reducing traffic injury deaths by half by 2030.

Bangladesh has a population of 163 million people, with one of the world’s highest population densities (1,240 people/km^2^) and a per capita gross domestic product of US$1,856.^13^ Like many LMICs, the country is deeply affected by the RTI epidemic but lacks a dedicated prehospital emergency medical system. Most victims currently receive care from untrained bystanders lacking appropriate medical equipment, and the country’s incredible traffic congestion can create significant delays in getting vehicles to and from crash scenes. The 2016 Bangladesh Health and Injury Survey, conducted by the Ministry of Health and Family Welfare, estimated more than 22,000 deaths and 3.4 million injuries on the roads in 2015.^14^ The majority of those RTIs involved young men whose death or serious injury often impacts entire families.

In other LMICs, community-based interventions have been created to provide prehospital services. Programs in Ghana and Uganda built on informal networks of prehospital providers, such as commercial drivers and police. Participants received trauma first-aid training, and follow-up interviews showed that this improved their ability to provide assistance to RTI victims.^15^^–^^18^ In landmine-prevalent areas of Iraq and Cambodia, community members were trained locally on advanced trauma life support, and these paramedics in turn trained networks of local laypeople as first responders. Their chain-of-survival trauma system proved effective in reducing deaths from landmines and other war-related injuries^19^^–^^22^ and decreasing mortality from RTIs.^23^ Based on the promise of earlier programs, in 2005, the World Health Organization recommended laypeople first responders as a low-cost way to improve trauma outcomes.^24^

When a traffic crash occurs in Bangladesh, there are almost always laypeople at the scene trying to help. However, without proper training or equipment, their efforts sometimes cause additional injuries. We felt that we could improve patient outcomes by harnessing this goodwill and providing training, equipment, and a way to rapidly mobilize first responders. Using other layperson first-responder programs as models and incorporating increasingly available mobile and telecommunication technologies, we developed TraumaLink to provide traffic injury victims with rapid and reliable access to first aid at the crash scene.

Using other layperson first-responder programs as models, we developed TraumaLink to provide traffic injury victims with rapid and reliable access to first aid at the crash scene.

High-quality data on RTIs are often unavailable in LMICs. The lack of reliable information to drive the creation and ongoing funding of effective countermeasures helps to perpetuate and exacerbate dangerous road conditions.^8^^,^^25^^–^^27^ Establishing a dense network of local volunteers helped us address the lack of comprehensive data on crashes and injuries by combining a sensitive surveillance system with a directed effort to collect and record all details within the first few hours after the completion of a crash response.

To better understand the program’s impact, we retrospectively analyzed data originally collected for quality monitoring and evaluation. We offer our descriptive analysis, focusing on key indicators of service performance and sustainability, as evidence for the model as a reliable and locally appropriate way to deliver on-scene trauma first aid through a community-based volunteer layperson first responder system. We used the Standards for QUality Improvement Reporting Excellence (SQUIRE) reporting guidelines in the creation of this article.^28^

## PROGRAM DESIGN AND IMPLEMENTATION

### Development of the Model

In January 2013, a team from Bangladesh and the United States with expertise in emergency medicine, public health, mobile health and technologies, medical education, and social organizing began investigating the feasibility of implementing a community-based trauma first-aid program using volunteers. We held numerous meetings with Bangladeshi organizations involved in road safety and volunteerism and spoke with academic researchers and local community members including government officials, police, fire services, public hospital staff, and religious leaders. These conversations were invaluable in developing and refining the operational model, and the open, trusting, and respectful relationships created in the process were essential in strengthening widespread and lasting support for the initiative.

We used the information and feedback we received to develop a program suited to local conditions in Bangladesh. On November 23, 2014, in the Daudkandi upazila (subdistrict), we launched the pilot of a model in which local community members are recruited and trained to act as layperson volunteer first responders treating traffic injury victims at the crash scene, free of charge. Volunteers are trained in crash scene management, basic trauma first aid, and mass casualty triage and provided with essential medical supplies kept in easily accessible locations.

Our call center, available through a dedicated emergency hotline phone number, uses a custom-designed graphic user interface (GUI) to rapidly connect injured victims with help. Operators receiving a call first collect information on where the crash occurred and how many patients have been injured. As soon as this information is entered into the GUI, the software uses an embedded algorithm to dispatch volunteers, prioritized by their proximity to the crash scene, by sending text messages to their mobile phones. Because this process is automated it leaves the operator free to notify police and fire services as needed.

Volunteers collect first aid kits en route to the crash scene and provide treatment using basic first aid and the principles of mass casualty triage. When patients are ready for transport, operators use the GUI to provide guidance on the nearest hospital capable of treating their injuries. Victims arrive there quickly using local transportation networks that include fire services, police, vehicles for hire, and bystanders.

### Needs Assessment

In June 2013, we conducted a semistructured needs assessment to better understand conditions in the proposed intervention area and to finalize the choice of our pilot community. We conducted interviews individually and in larger groups with a convenience sample of 273 key informants that included commercial drivers and small business and restaurant owners and patrons. These discussions and interviews were conducted in Bengali at 13 crash-prone locations along a 65-km stretch of the Dhaka-Chittagong Highway outside of Dhaka. These conversations aimed to assess the scope and nature of the RTI problem, formal and informal services available for injured victims, and opinions on whether the proposed model would be effective and desirable.

The dangers to road users on the major highways and lack of services for RTI victims were widely acknowledged, and people usually did not have ready access to phone numbers for police or fire services. They also cited lack of first aid training, fear of accidentally causing more harm to victims, and the limited availability of ambulance transport as barriers to victims receiving rapid and appropriate medical care. Many pointed out that private hospitals routinely turn away patients they think will not be able to afford the costs of treatment.

We also found a pervasive fear of interactions with the police and legal system based on police or bystanders sometimes accusing Good Samaritans of having caused the crash or trying to steal from victims. Those trying to assist injured patients can then be at risk of bystander violence, prosecution, or being called upon to provide witness testimony at the police station or in court.

These road users on the Dhaka-Chittagong Highway overwhelmingly expressed that having a single emergency hotline phone number, receiving training on providing basic trauma first aid, and having guidance on where to take injured patients would all be helpful, so we focused on rapidly connecting injured victims with first aid at the crash scene and transportation to the hospital.

During our needs assessment, and in meetings with experts and organizations involved in road safety, key informants highlighted the frequency with which RTI victims bleed to death from otherwise survivable injuries before reaching the hospital. A study conducted in Iraq and Cambodia observed that training lay first responders and paramedics in performing trauma care at the scene led to a significant decrease in the trauma mortality rate over 5 years.^20^ A study by the same group showed reduced mortality when patients received initial care from trained layperson first responders providing basic first aid.^21^ Thus, we focused on teaching basic first aid skills to community volunteers to have the greatest impact on trauma-related injuries and deaths.

Establishing a model to transport victims to health care facilities was another program priority. We quickly ruled out providing ambulance transport as part of the program due to cost and the area’s extreme traffic congestion. The first responder programs in Iraq and Cambodia utilized local transportation networks, and community-based transport systems have been used for obstetric emergencies in other settings.^29^^,^^30^

Because there was already an established culture of bystanders taking injured patients to the hospital, we asked communities for help with this aspect of the program. Fire services promised to prioritize calls from TraumaLink operators in dispatching their ambulances, expressing confidence that these calls for assistance would be appropriate and urgent. We also hoped that highway police and local police could use their vehicles to assist victims but recognized that this might be a challenge based on the fear of police identified during the needs assessment interviews.

Because there was already an established culture of bystanders taking injured patients to the hospital, we asked communities for help with patient transport.

Bangladeshi law enforcement agencies have traditionally focused on securing the crash scene and creating a legal case, rather than the rapid care of injured victims. As a consequence, well-intentioned bystanders are often discouraged from assisting people with injuries. To address this as a potential barrier to our operational model, we initially secured support for the program at the highest levels of the police administration; we then worked our way down the chain of command to form strong and ongoing ties with local and regional police departments.

### Recruiting and Training Volunteers

Before launching the service in a community, we organize multiple events to raise awareness about RTIs, foster discussions, and recruit potential volunteers. We also work with local religious and civic leaders, as well as the family members of those interested in volunteering, to address cultural and religious barriers that might discourage women from participating. Community members who express an interest in becoming volunteer first responders (VFRs) are interviewed by TraumaLink staff first. The staff decide who will receive training based on factors including where candidates live and/or work, personal experience with RTIs, prior efforts to treat or transport injured victims, motivations for wanting to serve, and feedback from other community members.

Individuals who are trained are all local citizens, most of whom live and/or work near the highway. They have to be at least 18 years old, but there are no minimum literacy or educational requirements. Although other programs have targeted training commercial drivers,^15^^–^^18^^,^^31^^–^^33^ we did not regard this as a viable option in Bangladesh, where any driver involved in a crash can become a target of violence from bystanders, so many drivers quickly flee the scene if they are well enough to do so.

VFR candidates receive 1 full day of training on providing basic trauma first aid from a Bangladeshi physician-trainer employed by TraumaLink, and other staff members assist those with limited literacy to ensure full participation. Classes are conducted locally and combine didactics with a strong emphasis on hands-on training, with class size limited to 15 students to ensure individual attention for all participants.

Our curriculum was based on materials originally developed by the primary investigator (JM) to teach community health workers in Uganda; it was further contextualized to Bangladesh and translated into Bengali with the assistance of Bangladeshi physicians. It focuses on the basics of providing trauma first aid, patient transport, and mass casualty triage with an emphasis on lifesaving skills that are easy to teach, learn, and perform. We also developed a professional-quality training video in Bengali that is provided to trainees in a high-definition version and a condensed version that can be downloaded and viewed on a mobile phone.

Students take a short written test of first aid knowledge before and after the class. The physician trainer also works closely with each of them throughout the day, allowing him to assess their knowledge of the course materials and procedural skills. No students failed the training course during the period of study, but area coordinators provided additional instruction and practice time for those who needed it. All students are encouraged to regularly review the training video and written materials and to practice these procedures with other people. Graduates receive a printed version of the training curriculum, a personal photo ID badge, and a reflective fluorescent vest featuring the TraumaLink logo; the badge and vest ensure that volunteers can be quickly identified in a crash scene and confer legitimacy and status.

In the first 6 years of the program, there were 11 geographic service expansions, bringing the program to a total of 135 km, spanning 11 upazilas (subdistricts) on 3 national highways. For each expansion of the service, we hold a launching ceremony featuring distinguished guests, community leaders, and new VFRs. At these events, all VFRs publicly take an oath to provide care to anyone in need and to refuse any financial or other type of compensation for their work. However, TraumaLink fully reimburses VFRs and area coordinators for any local travel expenses incurred while responding to crashes and transporting patients. To stay active in the program, VFRs are also required to undergo a half-day of retraining every 6–12 months.

### Program Overview

TraumaLink uses a dedicated emergency hotline number and a 24-hour call center staffed by paid full-time employees. A GUI, developed specifically for the program, features interactive and searchable maps of the catchment areas, densely populated with local landmarks. Before beginning operations, community members help staff identify these landmarks and their various, sometimes multiple, local names commonly in use. Staff collect exact GPS coordinates for each site on a smartphone and enter them, along with the local names, into the GUI. This allows for easy identification of the crash location even when the caller is not familiar with the community.

**Figure:**
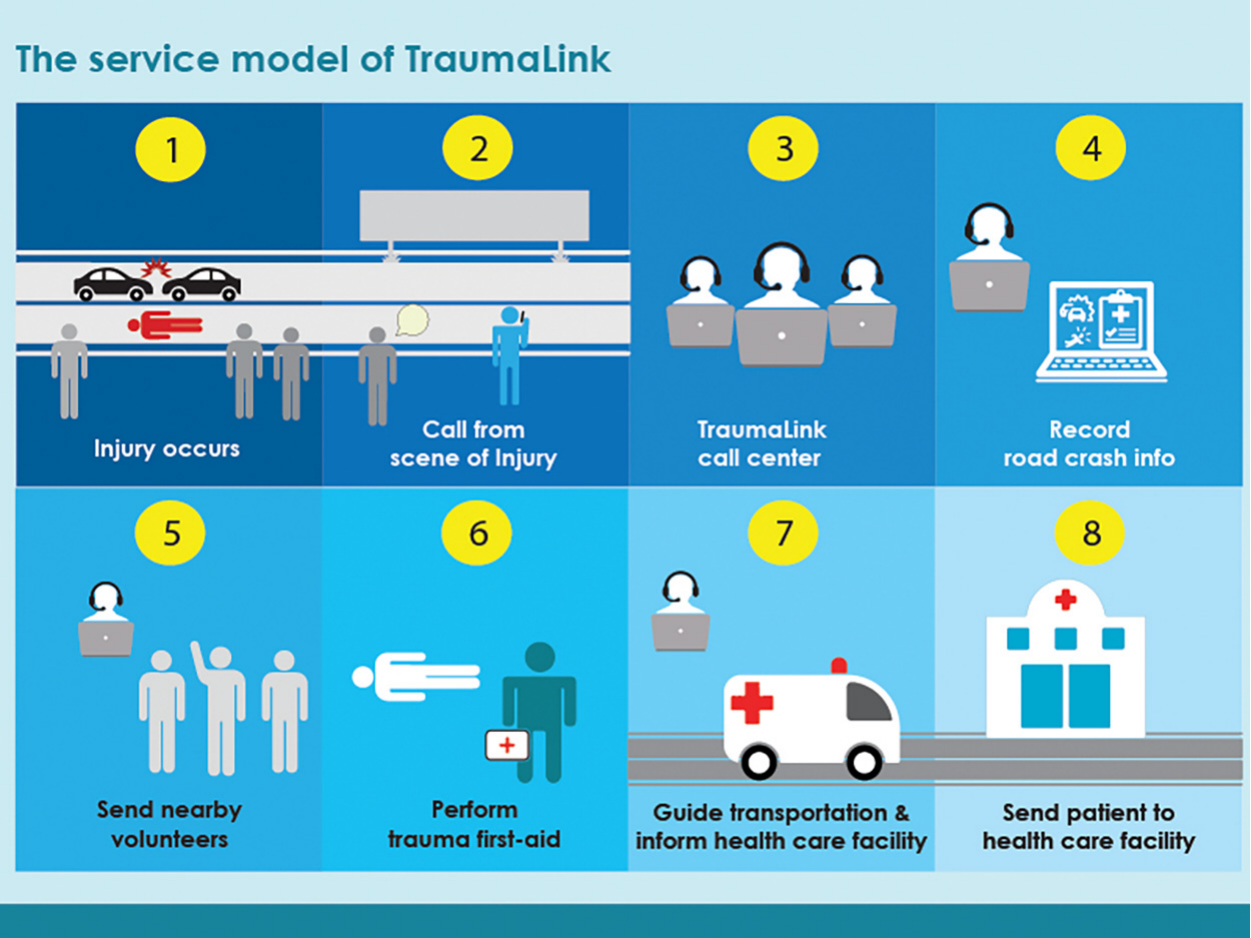
TraumaLink Service Model in Bangladesh © 2022 TraumaLink

Catchment areas are divided into operational zones roughly 1–2 km in length, based on local landmarks and the frequency of crashes in that area. All VFRs are assigned to a specific zone based on where they live and/or work and are dispatched to crashes on a rotating basis. For larger mass-casualty events, volunteers are also recruited from adjacent zones. TraumaLink provides and maintains locally sourced first aid supplies and stores them in locations that can be accessed 24 hours a day. Paid, full-time area coordinators supervise VFRs and provide local support and quality control; they also act as a vital link between the central office in Dhaka and local officials, volunteers, and community members.

At the request of the highway police, who have jurisdiction over the national highways, hotline operators routinely notify them of all crashes that might require their presence. The police provide scene management, protection, and legitimacy for the volunteers and transportation of victims to the hospital. Fire services are also activated as necessary to assist with rescue operations and ambulance transport.

Emergency hotline operators receiving a call first enter information on the crash location and number of injured patients. The GUI software then uses an embedded algorithm to generate text messages, dispatching an appropriate number of VFRs prioritized by their proximity to the crash scene. After patients are treated at the accident scene, hotline operators make a rough assessment of the severity of their injuries based on information from the VFRs, and using triage guidelines developed for the program. The call center software also contains a registry of public hospitals categorized by the severity of traumatic injuries they can manage, and operators use this information as needed to direct patients to the nearest appropriate facility. We default to sending patients to public hospitals to avoid potential delays in care resulting from private hospitals refusing them admission and to protect VFRs from having to make potentially financially consequential decisions for incapacitated patients.

To reduce the risks of emotional trauma in the VFRs, area coordinators initially organized monthly meetings to discuss patient care experiences. This approach was based on an understanding that first responders of all types often find it easiest and most helpful to talk about their traumatic experiences with other first responders. Additionally, these meetings provide a forum to freely exchange information and ideas, allowing us to improve our service offering and to rapidly identify and address any operational issues. As the service has expanded and matured these meetings have gradually become less frequent, but area coordinators or other volunteers still regularly spend time with VFRs after difficult calls, providing them with emotional support. VFRs’ enhanced social status, strong community backing, and palpable sense of helping their fellow citizens are also likely protective.

Before each expansion of the program into a new community, we advertise our services and hotline number through community engagement events and various types of local media. Volunteers and staff also regularly spend time in the community, educating people about the service and encouraging them to program the hotline number into their phones. These campaigns are continued after the service launch.

## DATA COLLECTION AND ANALYSIS

For quality monitoring and evaluation of the program as well as to determine the program’s impact on RTIs, after completion of each incident response, call center operators follow up by phone with volunteers and/or area coordinators to gather any missing information such as types of road users, victim demographics, and clinical information including patient dispositions.

Local Bangladeshi staff assembled and checked data from the first 6 years of operations—from November 23, 2014, through November 22, 2020—for accuracy and completeness. Results were translated into English and the de-identified data were made available for analysis. All information was collected in a Microsoft Excel spreadsheet.

We chose our data analyses to reflect the reliability and efficiency of TraumaLink operations, as proxies for improved clinical outcomes, since we lacked detailed clinical data on our patients. However, the benefits of prehospital trauma care are well established.^10^^,^^34^ We measured the percentage of calls receiving a response, the interval between the crash and the arrival of a VFR or area coordinator, the percentage of patients for whom transportation to the hospital could be found, the interval between the crash and arrival at the hospital, and the alignment of prehospital injury-severity assessments with patient dispositions. We also examined volunteer retention as a measure of sustainability and analyzed patient demographics to better understand the target population and broader societal impacts of the program. Because there were no dedicated prehospital first aid services in place before the intervention, improvements in the provision of care were attributed to TraumaLink’s implementation. We did not study long-term trends in crash occurrences, as crash prevention was not a primary goal of the program.

### Ethics Approval

We obtained internal review board approval through the Office of Human Research Administration at the Harvard T.H. Chan School of Public Health (Protocol # IRB15-3995). Because there was no access to data with unique identifiers or links/codes to personal identifying information, the project was granted a “not human subjects” determination.

### Patient and Public Involvement

The public was actively involved from the program’s inception and participated in the initial needs assessment and stakeholder discussions, development and refinement of the operational model, and choice of the pilot project location. Based on stakeholder feedback, we focused on sustainably delivering rapid and reliable care at the crash scene, which guided priorities for data collection and analysis. In each geographic expansion of the program, community leaders help to suggest and vet potential volunteers and remain actively involved throughout implementation and after the service launch. We regularly provide the compiled data, including maps of crash locations, to the highway police and local policy makers.

## RESULTS

TraumaLink, legally incorporated as a social enterprise in Bangladesh in August 2013, formally began operations in November 2014 on a 14-km stretch of the Dhaka-Chittagong Highway in Daudkandi, Comilla District.

Services ran without interruption and with only minor adjustments to the GUI and field operations necessary after rollout. In March 2020, infection control measures were instituted for the coronavirus disease (COVID-19) pandemic and, while demand for the service remained high, no staff or volunteers left the program or became ill in the course of their duties. By the end of the study period, operations had been expanded to a total of 135 km of coverage on 3 national highways and complete data were available for all crash responses.

By 2020, the program had been expanded to a total of 135 km of coverage on 3 national highways.

### Emergency Response

**FIGURE 1:**
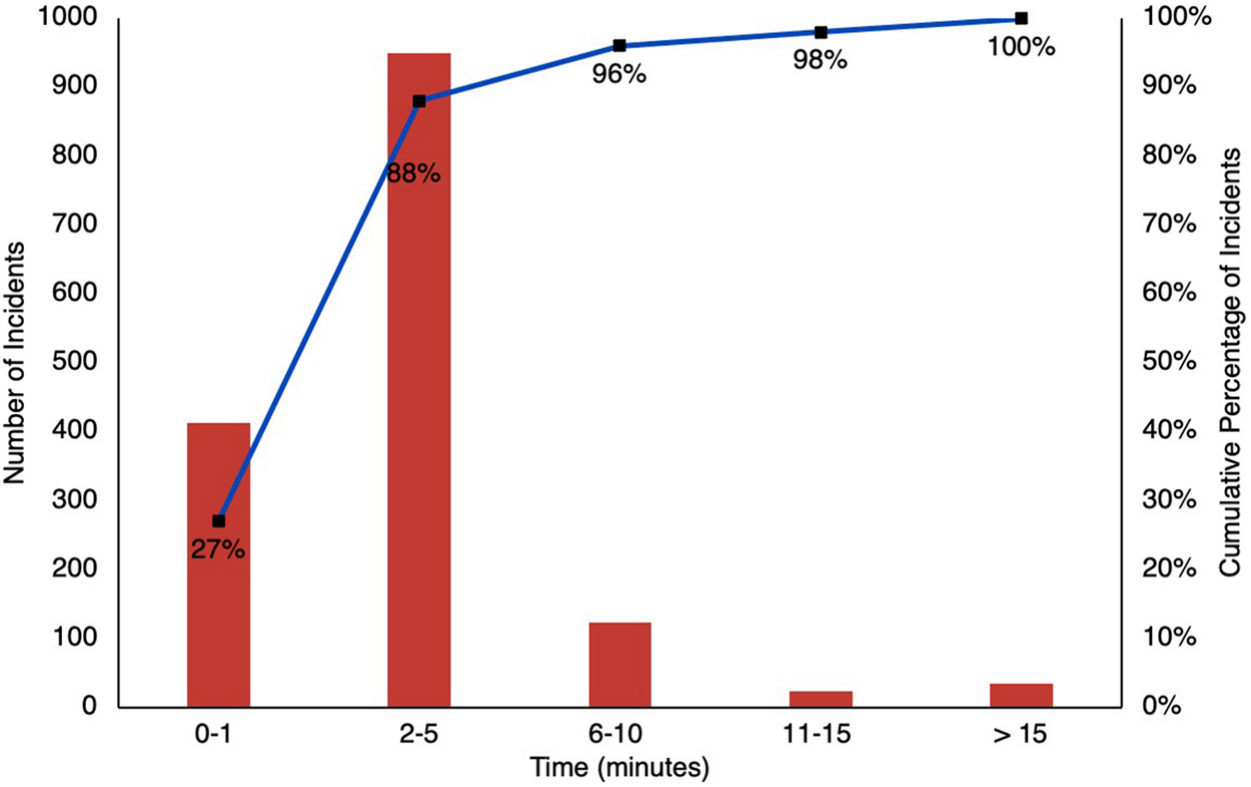
Summary of Times Between Crash and Arrival of Volunteer or Area Coordinator Using TraumaLink in Bangladesh

**FIGURE 2:**
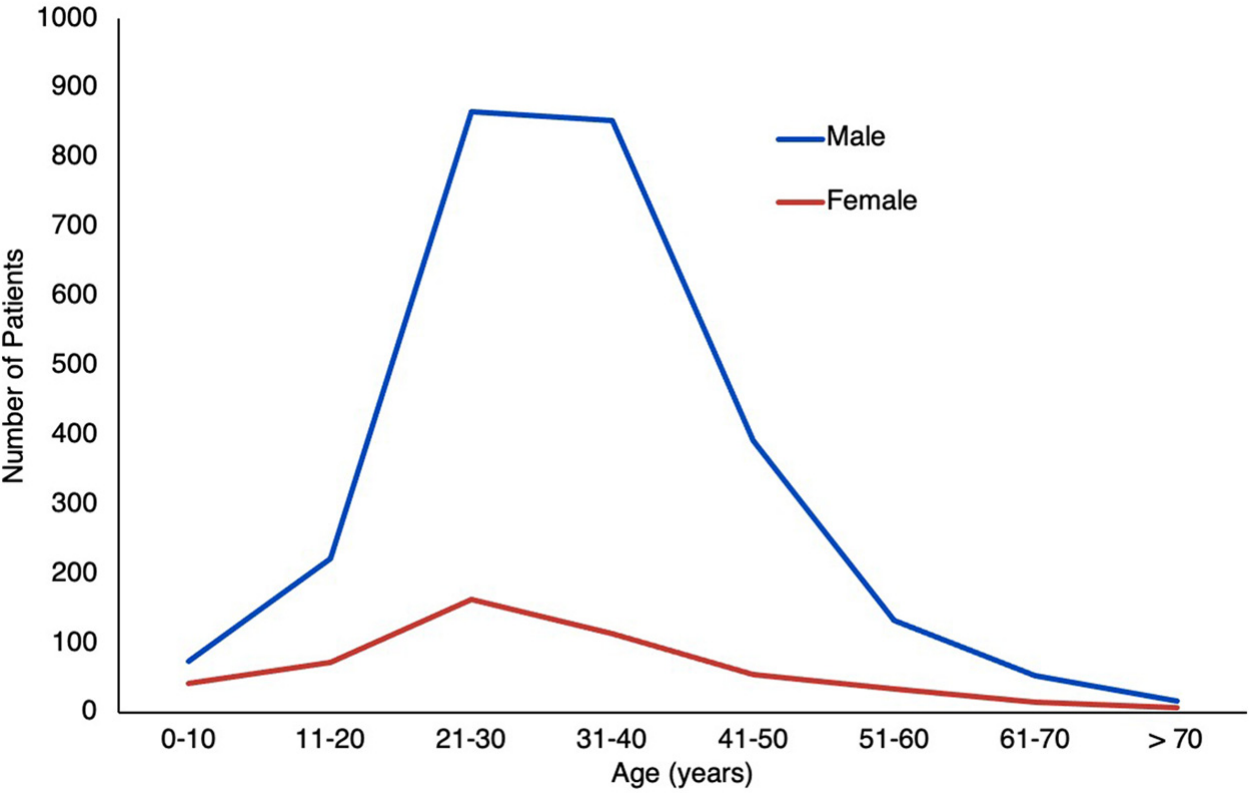
Age and Gender of Treated Patients Using TraumaLink in Bangladesh

During the study period, TraumaLink provided free care to 3,119 patients involved in 1,544 crashes, with VFRs or area coordinators initiating the call in 1,463 (95%) of those crashes. All calls to the hotline received an emergency response, and arrival times to the incident scene were 5 minutes or less from the time of the crash in 1,363 (88%) incidents (Figure 1). More than half of the victims (1,721; 55%) were men aged between 21 and 40 years (Figure 2).

### Patient Injury Assessments

Based on clinical information from the responding volunteers, call center operators made basic prehospital injury severity assessments for each patient to help prioritize them for transport and direct them to the nearest appropriate hospital. Among our patients, 1,180 (38%) were assessed with light injuries; 1,245 (40%) with moderate injuries; and 694 (22%) with severe injuries.

Overall, 1,220 (39%) patients were treated at the scene and sent home and 1,309 (42%) were treated at the scene and then sent to the hospital for further care; and 590 (19%) patients were immediately taken to the hospital without receiving treatment at the scene. Most of these 590 were victims of severe injuries who were stabilized en route; however, some were victims who had less serious injuries but were involved in mass-casualty incidents where providing treatment to every patient at the scene was not feasible. Of the 1,899 patients transported to the hospital, 1,078 were discharged home after treatment and 821 were admitted for further care.

**FIGURE 3:**
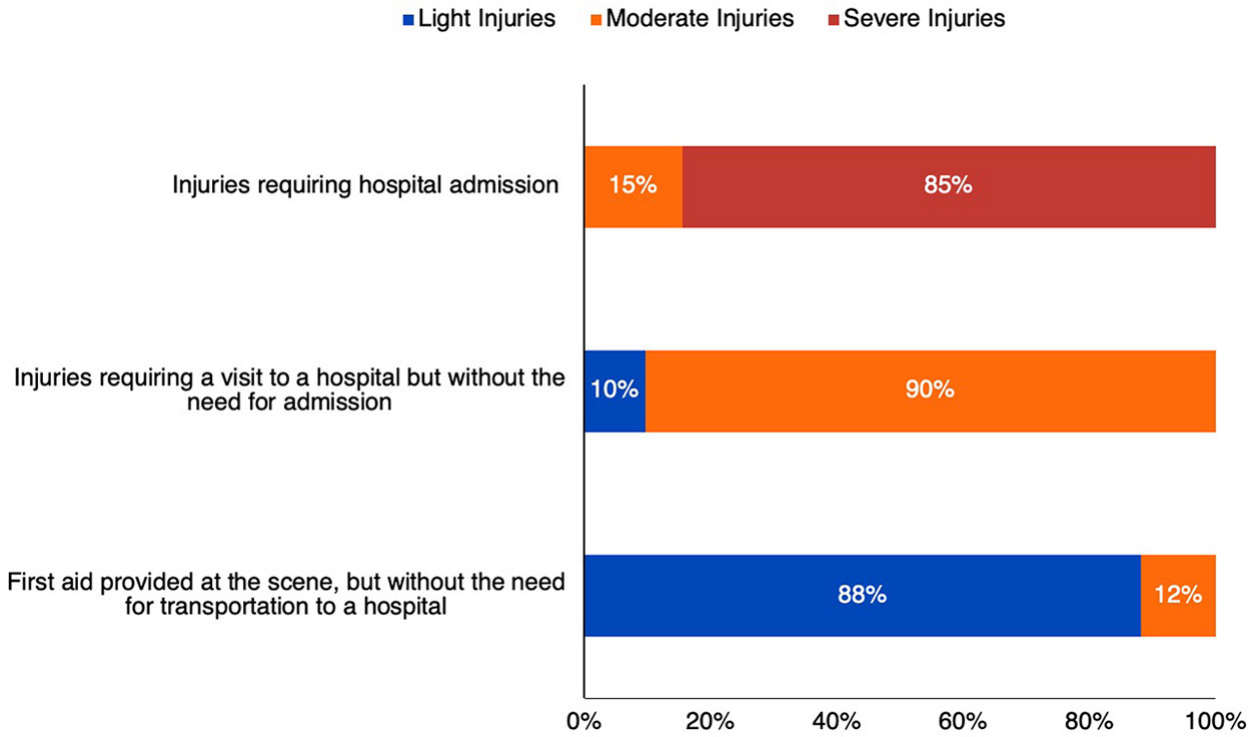
Prehospital Injury Severity Assessments and Patient Dispositions for Patients Treated by TraumaLink

Injury-severity assessments correlated closely with patient dispositions, reflecting the accuracy of on-scene triage decisions (Figure 3). Of the 1,220 patients treated at the scene and sent home, 1,076 (88%) were determined to have light injuries. Among 1,078 patients transported to the hospital but discharged home after treatment, 974 (90%) had moderate injuries, and severely injured patients accounted for 694 (85%) of the 821 victims admitted to the hospital. No patients assessed with light injuries were admitted to the hospital, and no one determined to have severe injuries was discharged home.

### Patient Transport

**FIGURE 4:**
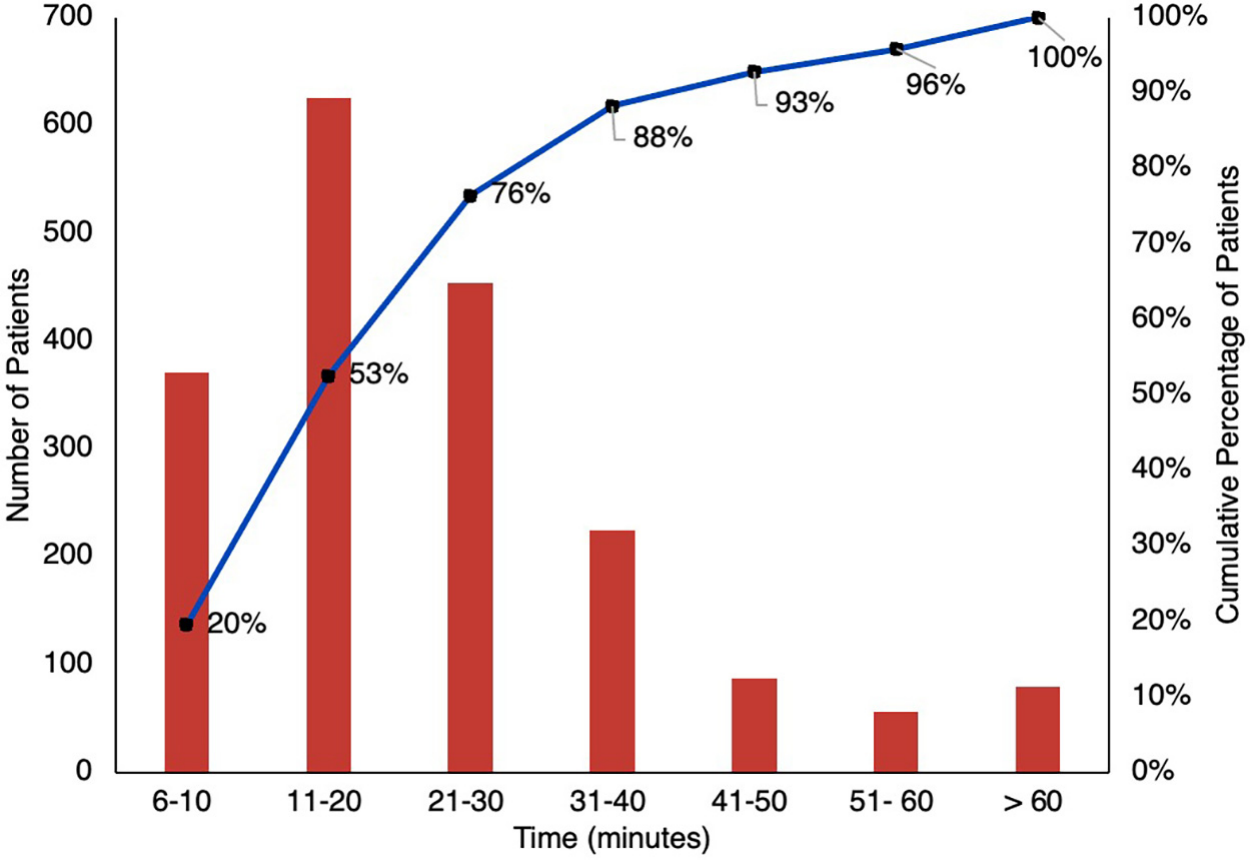
Summary of Times Between Crash and Arrival of Patient at the Hospital Using TraumaLink in Bangladesh

Volunteers and area coordinators found transportation for all 1,899 victims requiring transfer to the hospital. The most, 371 (20%), were taken in a fire service ambulance, whereas 340 (18%) were taken by "CNGs"—motorized 3-wheel vehicles for hire that are widely used for local travel. All victims taken to the hospital were accompanied, most frequently by VFRs and/or area coordinators (1,115; 59%) or fire service personnel (359; 19%). In instances where bystanders transported patients from the scene before first responders arrived, those responding immediately proceeded to the hospital to tend to, and advocate for, the victims until a family member or another responsible party arrived. Most patients (1,451; 76%) arrived at the hospital within 30 minutes of the time of the crash (Figure 4).

Although VFRs’ training is focused on RTIs, as the program matures in a community, VFRs also begin getting calls for other types of trauma, such as farming injuries, but these were not included in the current analysis.

### VFR Retention

A total of 599 volunteers (536 men and 63 women) passed the training course and were registered as VFRs. There were no instances of police or community members harassing volunteers or interfering with their work, and 545 VFRs (489 men and 56 women) were still actively working at the end of the study period. Of the 54 people leaving the service, 41 moved out of the area for a new job and/or marriage, 10 resigned (all during the 18-month pilot) because they had not been called for any incidents, 2 became too busy to continue, and 1 died. Among other benefits, this strikingly low VFR turnover yielded a pool of experienced, engaged, and unfailingly reliable volunteers that decreased the demands on area coordinators and allowed these paid staff to cover much broader service areas than we initially anticipated.

## DISCUSSION

Our initial needs assessment found that people reported fear of interactions with police and the judicial system as a major impediment to being Good Samaritans responding to traffic accidents. Ambulance transport for RTI victims in Bangladesh is uncommon,^35^ and patients are frequently turned away from private hospitals out of concern for their ability to pay for services.

However, individuals in our study highlighted well-established cultures of bystanders providing assistance, primarily through calling police, finding transportation to the hospital, and/or notifying family members. Most stakeholders in these discussions advocated for a single hotline number, training on basic trauma management skills, guidance on transporting injured patients, and protections from police harassment and entanglement in the legal system. These findings were similar in many respects to those of a recent qualitative study of barriers to bystander assistance of trauma victims in Delhi, India.^36^

The introduction of TraumaLink streamlined and augmented the process of mobilizing and organizing prehospital services for RTI victims. Previously, police and fire services were notified of crashes through bystanders calling the stations, if they had the numbers available. We instead instituted a hyperlocal emergency response system, with VFRs positioned to quickly learn about and respond to incidents. The proximity of the volunteers and their dominant role in initiating emergency responses enabled our rapid response times; in the middle of the night, it was often a volunteer who heard the crash, responded to the scene, and formally activated the call center. The VFRs also became well known in their communities and were frequently called first by witnesses to a crash. They would then notify operators on their way to the scene.

The introduction of TraumaLink streamlined and augmented the process of mobilizing and organizing prehospital services for RTI victims.

Although TraumaLink was a new initiative, communities showed a remarkable level of dedication and commitment to the service, clearly evidenced in the 100% incident response rate. Many more community members offered to act as VFRs than we had positions available, and turnover was low. There were no prank calls or instances where volunteers violated the code of conduct. We also found transportation for every patient requiring transfer to the hospital, with the community’s willingness to provide this assistance further reflected in 96% of patients arriving within the “golden hour.” Additionally, the program improved relationships between police and the community and elevated the visibility and status of women through their work as volunteers.

The high number of young men among our patients reflects their predominance on the roads and has been noted in studies of RTI victims elsewhere.^11^^,^^16^^,^^23^^,^^37^^,^^38^ Since many of these men are family breadwinners, RTIs can have profound economic ripple effects on their relatives and communities, highlighting the role of unsafe roads in creating new or worsening poverty.^37^^,^^38^

Based on anecdotal reports from patients, first responders, and hospital staff, rapid bleeding control was an important aspect of our service offering (Box). This was also the most frequently used skill in the Ghana and Uganda projects and played a central role in the programs in Iraq and Cambodia focused on treating victims of landmine injuries.

BOX **TraumaLink Patient Testimonials^a^**

*I got into road crash injury and TraumaLink provided the first aid service to me. They immediately stopped my bleeding and bandaged my injured part. Later, when I went to Dhaka’s Medical Hospital for treatment, the doctors told me that TraumaLink did a very good job. If they did not stop the bleeding, the injury would have got extremely severe otherwise.*

*On a road crash I got injured on different parts of my body and I was bleeding. Then, TraumaLink’s volunteer arrived at the scene and quickly provided me with emergency first aid treatment. TraumaLink helped me by providing emergency treatment at the right time. I survived a massive crash. TraumaLink provides their service free of cost. I would like to thank them for their work.*

*It was one foggy winter night, when I, along with my wife and 9-month-old child, got into a road crash. Right at that moment, I felt so helpless. Then within 1 to 2 minutes TraumaLink’s volunteer stepped into the incident scene and very promptly provided the emergency first aid service for us. I never imagined in my life that I would get such a service from anyone.*

^a^Excerpted from interviews conducted in Bengali and translated into English.

Although most first responders and call center operators did not have previous medical training, their assessments of injury severity aligned closely with patient dispositions, but this association may not be as strong as it initially appears. In certain crash responses, especially those involving multiple casualties or severely injured victims, TraumaLink operators would collect any missing information, including patient dispositions, only after all patients had been treated and transported from the scene. However, all patients determined to have severe injuries were admitted based on independent assessments by hospital staff.

### Limitations

In this retrospective analysis of operational data, we lacked detailed clinical information on individual patients and did not have baseline data available for comparison. These limitations made it difficult to accurately quantify the program’s clinical and cost-effectiveness, and more rigorous evaluation of both is needed as we move forward.

Although we provided training and guidance on transporting badly injured patients to the nearest trauma center, in practice the majority of patients were taken directly to the closest available facility—commonly small government hospitals with rudimentary emergency departments and no surgical capabilities. This finding was not unexpected; we recognized that it was unrealistic to expect Good Samaritans to routinely bypass a local hospital and undertake a prolonged trip to another facility. Furthermore, first responders had little ability to monitor and stabilize patients during transport to a more advanced hospital, a trip that can sometimes take hours even in an ambulance.

We were encouraged by the participation of women in the program, although we had hoped for more balanced gender representation in our pool of VFRs. Some of these women were among our most active volunteers, and all were treated with a great deal of trust and respect by their communities. Of the 7 who left the service, 6 moved away for work and/or marriage and 1 died. None left because of harassment, lack of family or community support, or dissatisfaction with the work.

Although the operational model worked well, we had less success in achieving financial sustainability. We relied heavily on independent funding and award money to sustain the project as we sought additional support through sponsorships, corporate social responsibility funding, and donations. Partnering with an established and well-respected company in Bangladesh gave us a strong foundation, but we still had more difficulty bringing in local support than we had anticipated. Many organizations appreciated our work, but our small scale of operations could make it difficult for them to justify using limited marketing and corporate social responsibility funds. We also investigated for-profit first aid training for local businesses and nongovernmental organizations but found little interest in these courses. As we expanded the scale of our services, however, we gained the attention of and began to draw more support from larger Bangladeshi businesses as well as international companies and nongovernmental organizations. We hope that such support will be an important part of our plan for long-term sustainability.

## CONCLUSION

The strong community support and rapid, reliable volunteer responses that characterize TraumaLink suggest that this model could be expanded throughout Bangladesh. While each setting offers unique challenges, many of the dangers Bangladeshi road users face are found throughout LMICs, and this simple, effective, and easily scalable model could be readily modified for other LMICs facing similar challenges.

This community-based prehospital care network can also serve as an important resource in the immediate aftermath of natural or man-made disasters. A group of trained and equipped first responders, who are well-known within their communities and have experience managing mass casualty incidents, can be an invaluable asset at a time when outside resources are lacking and timely care is of the utmost importance. We hope that over time this simple program designed for RTI victims can also serve as a stepping-stone to a more advanced emergency medical system offering higher-level training and a broader range of services.

## Notes

**Peer Reviewed**

**First published online:** July 28, 2022.
